# Prognostic Value of Quantitative [^18^F]FDG-PET Features in Patients with Metastases from Soft Tissue Sarcoma

**DOI:** 10.3390/diagnostics11122271

**Published:** 2021-12-04

**Authors:** Gijsbert M. Kalisvaart, Willem Grootjans, Judith V. M. G. Bovée, Hans Gelderblom, Jos A. van der Hage, Michiel A. J. van de Sande, Floris H. P. van Velden, Johan L. Bloem, Lioe-Fee de Geus-Oei

**Affiliations:** 1 Department of Radiology, Leiden University Medical Centre, 2333 ZA Leiden, The Netherlands; W.Grootjans@lumc.nl (W.G.); F.H.P.van_Velden@lumc.nl (F.H.P.v.V.); J.L.Bloem@lumc.nl (J.L.B.); L.F.de_Geus-Oei@lumc.nl (L.-F.d.G.-O.); 2 Department of Pathology, Leiden University Medical Centre, 2333 ZA Leiden, The Netherlands; J.V.M.G.Bovee@lumc.nl; 3 Department of Medical Oncology, Leiden University Medical Centre, 2333 ZA Leiden, The Netherlands; A.J.Gelderblom@lumc.nl; 4 Department of Surgical Oncology, Leiden University Medical Centre, 2333 ZA Leiden, The Netherlands; J.A.van_der_Hage@lumc.nl; 5 Department of Orthopaedic Surgery, Leiden University Medical Centre, 2333 ZA Leiden, The Netherlands; M.A.J.van_de_Sande@lumc.nl; 6 Biomedical Photonic Imaging Group, University of Twente, 7522 NB Enschede, The Netherlands

**Keywords:** metastatic soft tissue sarcoma, [^18^F]FDG-PET, prognosis

## Abstract

Background: Prognostic biomarkers are pivotal for adequate treatment decision making. The objective of this study was to determine the added prognostic value of quantitative [^18^F]FDG-PET features in patients with metastases from soft tissue sarcoma (STS). Methods: Patients with metastases from STS, detected by (re)staging [^18^F]FDG-PET/CT at Leiden University Medical Centre, were retrospectively included. Clinical and histopathological patient characteristics and [^18^F]FDG-PET features (SUVmax, SUVpeak, SUVmean, total lesion glycolysis, and metabolic tumor volume) were analyzed as prognostic factors for overall survival using a Cox proportional hazards model and Kaplan–Meier methods. Results: A total of 31 patients were included. SUVmax and SUVpeak were significantly predictive for overall survival (OS) in a univariate analysis (*p* = 0.004 and *p* = 0.006, respectively). Hazard ratios (HRs) were 1.16 per unit increase for SUVmax and 1.20 per unit for SUVpeak. SUVmax and SUVpeak remained significant predictors for overall survival after correction for the two strongest predictive clinical characteristics (number of lesions and performance status) in a multivariate analysis (*p* = 0.02 for both). Median SUVmax and SUVpeak were 5.7 and 4.9 g/mL, respectively. The estimated mean overall survival in patients with SUVmax > 5.7 g/mL was 14 months; otherwise, it was 39 months (*p* < 0.001). For patients with SUVpeak > 4.9 g/mL, the estimated mean overall survival was 18 months; otherwise, it was 33 months (*p* = 0.04). Conclusions: In this study, SUVmax and SUVpeak were independent prognostic factors for overall survival in patients with metastases from STS. These results warrant further investigation of metabolic imaging with [^18^F]FDG-PET/CT in patients with metastatic STS.

## 1. Introduction

Approximately 14% of patients with a soft tissue sarcoma (STS) present with metastatic disease [1]. Additionally, up to 34% of high-grade STS patients develop distant metastases within 5 years after resection of localized STS [2,3]. While several studies show an improvement in the survival of patients with metastatic STS over the last decades, the two-year survival rate remains less than 50% [4,5,6]. Indeed, treatment of these patients is complex due to the heterogeneous and aggressive nature of these tumors. Generally, therapies can consist of combinations of surgery, radiotherapy, and systemic treatment. Personalized decision making is important in designing treatment strategies, and a multitude of parameters is used for this purpose [7]. Prognostic factors play an important role among these parameters, and several studies have identified a group of characteristics that is associated with prognosis in these patients [4,5,6,8,9,10]. These studies strike the consensus that patient age, performance status, disease-free interval, and histological subtype are strong predictors for overall survival (OS). Nevertheless, stratification of patients on an individual level remains a difficult challenge and requires further insight in the link between tumor characteristics and prognosis.

The use of ^18^F-fluorodeoxyglucose ([^18^F]FDG) positron emission tomography (PET) for the characterization of malignant lesions is widely studied [11]. In STS patients, [^18^F]FDG-PET imaging is regularly performed for (re)staging and follow-up [12]. Furthermore, in metastatic STS, specifically, a recent study has shown value of [^18^F]FDG-PET in monitoring the response to systemic treatment [13]. The uptake of [^18^F]FDG, as expressed by the standardized uptake value (SUV), reflects the degree of glucose metabolism of a lesion. High [^18^F]FDG-uptake has shown to be connected to increased tumor aggressiveness in many STS subtypes. Especially in localized STS, several [^18^F]FDG-PET features, such as maximum SUV (SUVmax), peak SUV (SUVpeak), metabolic tumor volume (MTV), and total lesion glycolysis (TLG), are found to have significant prognostic value [14,15]. Moreover, in other tumor types, these parameters have shown to be predictive for survival in metastatic disease and demonstrated to be valuable for the personalization of treatment decisions [16]. While the metabolic properties of lesions, as indicated by quantitative [^18^F]FDG-PET features, might also provide valuable information for the prognosis of metastatic STS patients, no literature is readily available on the correlation between these features and survival. In the current study, we assessed the prognostic value of quantitative [^18^F]FDG-PET features in patients diagnosed with metastases from STS.

## 2. Materials and Methods

### 2.1. Patients

Patients with biopsy-proven STS, who underwent a [^18^F]FDG-PET/CT for (re)staging purposes on which metastatic disease was detected, were retrospectively included. Metastatic disease was defined as radiological evidence of systemic spread of tumor outside the primary tumor bed. Patients with GISTs (gastrointestinal stromal tumor) and primary uterine or retroperitoneal sarcomas were excluded to guarantee a relatively homogeneous population regarding tumor biology and treatment. Patients who received radiotherapy or systemic therapy for metastatic disease before [^18^F]FDG-PET/CTs acquisition were also excluded. Furthermore, all [^18^F]FDG-PET/CTs had to be performed between January 2017 and January 2021 at Leiden University Medical Center, which is a tertiary referral center for sarcoma care. Requirement to obtain patient consent was waived by the local ethical board, since clinical data were retrospectively collected and pseudo-anonymized.

### 2.2. Patient Characteristics

Clinical and histopathological characteristics, which were reported as independent prognostic factors of survival in previous studies, were collected for all included patients (Table 1). Primary tumor location was categorized based on the results of Lochner et al. to realize substantial group sizes for analysis [4]. Primary tumor localization in the deep trunk or upper extremity was categorized as high risk for impaired survival, while other locations were considered to be low risk. Since some patients were diagnosed with metastatic disease at first diagnosis of STS, the disease-free interval after resection of the primary tumor was not analyzed as a continuous variable but categorized in three groups based on the methods of Italiano et al. and Lochner et al. [4,5]. Patients who were diagnosed with metastatic disease at first diagnosis were categorized as ‘synchronous’. Patients who developed metastases after resection of the primary tumor were dichotomized around the median number of months of the disease-free interval. Reported World Health Organization (WHO) scale and the Fédération Nationale des Centres de Lutte Contre le Cancer (FNCLCC) system scores were collected from patient files and used for analysis of performance status and tumor grade, respectively [17].

### 2.3. [^18^F]FDG-PET/CT

All scans were acquired on a digital Vereos PET/CT scanner (Philips Healthcare, Best, The Netherlands) according to the most recent European Association for Nuclear Medicine (EANM) procedure guidelines for tumor imaging [18]. The PET/CT scanner was accredited by the Research4Life (EARL) initiative for quantitative PET/CT imaging. Patients fasted at least 6 h before imaging and were hydrated with 500 mL of water. [^18^F]FDG was administered 60 min before the acquisition of the PET scan. A low-dose CT scan (52 mAs, 120 kVp) was acquired prior to PET acquisition for the purpose of attenuation correction and anatomical reference. Standard [^18^F]FDG PET/CT scans were acquired from the skull base to mid-thigh or toes depending on the location of the primary tumor. Image acquisition time was 2 min per bed position. Image reconstruction was performed using a blob-based 3D iterative reconstruction algorithm (blobTOF; 3 iterations and 9 subsets) followed by a 5.5 mm full-width at half maximum (FWHM) post-reconstruction Gaussian filter. The image voxel size was 4 × 4 × 4 mm^3^. After reconstruction, all PET images were expressed in SUV by normalizing voxel radioactivity concentrations [kBq·mL^−1^] to the injected dose of [^18^F]FDG [MBq] and the patient’s body weight (kg).

### 2.4. [^18^F]]FDG-PET Features

**Figure 1:**
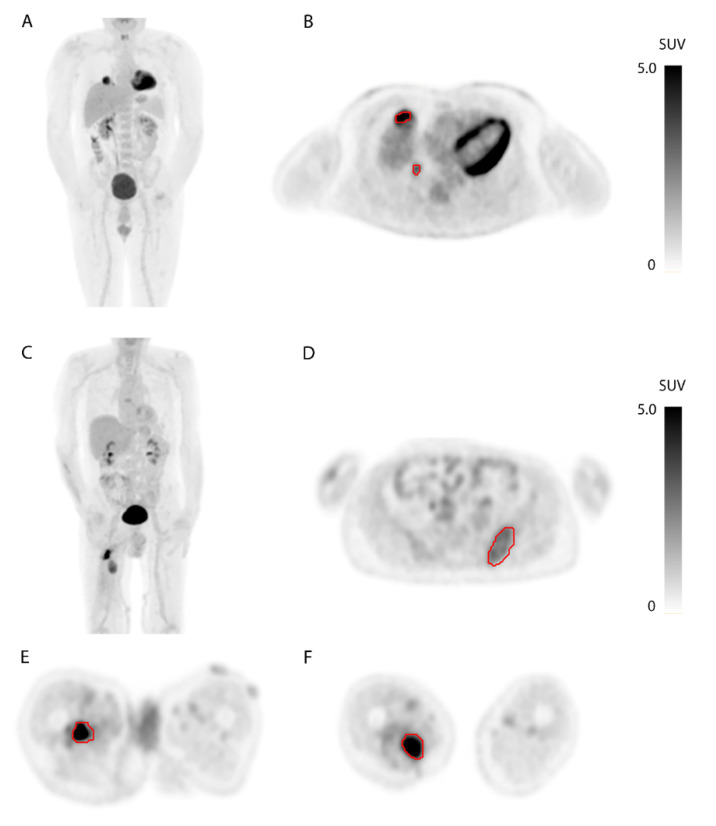
(**A**,**B**): [^18^F]FDG-PET of a man 8 months after resection of a primary undifferentiated soft tissue sarcoma (grade 3). (**A**): Coronal view of a maximum-intensity projection (MIP) showing two metabolically active lesions in the right lung. (**B**): Axial plane showing two metastases in the right middle lobe and their VOIs outlined in red. (**C**–**F**): [^18^F]FDG-PET of a man with three known tumor locations 6 months after resection of a primary myxofibrosarcoma (grade 2). (**C**): Coronal projection of a MIP showing two FDG-avid lesions in the right upper leg. (**D**): Axial plane showing a histologically proven metastasis in the left iliac bone and the corresponding VOI outlined in red. (**E**): Axial plane showing a tumor lesion and the corresponding VOI outlined in red in the adductor compartment of the right thigh just cranial of the primary tumor bed. (**F**): Axial plane showing local recurrence and corresponding VOI outlined in red in the adductor compartment of the right thigh.

Image analysis was performed using Philips Intellispace Portal software v10.1 (Philips Healthcare, Best, The Netherlands). Segmentation of all STS lesions was performed using an adaptive threshold algorithm. A segmentation threshold of 50% of the SUVpeak corrected for local background was used (Figure 1) [19]. After image segmentation, the resulting volumes of interest (VOIs) were used to calculate relevant uptake parameters in the PET images. For VOIs that covered normal tissue surrounding tumor lesions due to relatively high FDG uptake (e.g., heart tissue or urinary bladder), manual adjustment was performed to exclude normal tissue from the VOI. For every patient, the SUVmax and SUVpeak were calculated on the lesion with the highest SUVmax and with the highest SUVpeak, respectively. The SUVmax is defined as the voxel with the highest intensity within a tumor. The SUVpeak is defined as the largest mean value of a 1 cm^3^ sphere positioned within a tumor. Furthermore, SUVmean, MTV, and TLG were calculated for all lesions combined per patient (whole body). The SUVmean is defined as the mean of all pixel values within all tumor lesions in a patient. The MTV is defined as the sum of the volume of all tumor lesions in a patient. The TLG is defined as the sum of the products of the SUVmean and its corresponding MTV of each lesion.

### 2.5. Statistical Analyses

An univariate Cox proportional hazard model was used to determine the predictive value of clinical parameters and [^18^F]FDG-PET features for OS. No analysis of histologic subtypes was performed in this heterogeneous population due to the small number of patients per subtype and previous studies reporting variable histologic subtypes to be correlated to survival. Due to the limited cohort size, not all variables were tested in the multivariate cox analysis. Therefore, multivariate Cox analysis was first performed using the 2 strongest prognostic clinical factors. Subsequently, the prognostic value of adding [^18^F]FDG-PET features that were significant in univariate analysis was determined for each [^18^F]FDG-PET feature separately. The [^18^F]FDG-PET features that significantly added prognostic value to clinical parameters were stratified through the median and the Kaplan–Meier method, and log-rank test were used to estimate survival for the different groups. Statistical significance was defined as *p* < 0.05. The analysis was performed with IBM SPSS v.25 (IBM Corp., Armonk, NY, USA).

## 3. Results

### 3.1. Patients and Follow-Up

A total of 31 patients were included in this study, and segmentation of all STS lesions was performed (Figure 1). Patient characteristics are shown in Table 1. Median follow-up in survivors was 32 months. The two-year survival rate was 37%.

### 3.2. Univariate Analysis

**Figure 2:**
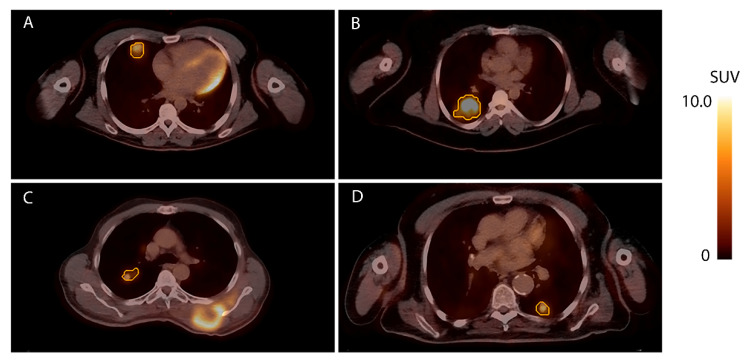
Examples of lung metastases with different metabolic characteristics. Axial planes of [^18^F]FDG-PET/CTs of four patients with lung metastases are shown with the corresponding VOIs outlined in orange. (**A**): A metastasis with a SUVmax of 6.1 detected 22 months after resection of an undifferentiated soft tissue sarcoma in the right deltoid muscle. (**B**): A metastasis with a SUVmax of 9.0 detected 34 months after resection of an undifferentiated soft tissue sarcoma in the right gluteus maximus. (**C**): A metastasis with a SUVmax of 5.2 detected synchronous with a myxofibrosarcoma originating from the left thoracic wall. (**D**): A metastasis with a SUVmax of 7.2 detected synchronous with local recurrence of a leiomyosarcoma in the right lower leg.

The number of lesions was the only clinical parameter that was significantly predictive for survival in this population (*p* = 0.006) (Table 2). Furthermore, analysis of the [^18^F]FDG-PET features showed SUVmax and SUVpeak to be significantly predictive for survival (*p* = 0.004 and 0.006, respectively) (Figure 2). Hazard ratios (HRs) were 1.16 per unit increase for SUVmax and 1.20 per unit increase for SUVpeak in univariate analysis.

### 3.3. Multivariate Analysis

The two strongest predictive clinical parameters were the number of lesions and the performance status. Adding SUVmax and SUVpeak separately to the multivariate model with these clinical parameters showed that both SUVmax and SUVpeak significantly improved the prediction (*p* = 0.005 and 0.004, respectively), independent of these clinical parameters. HRs were 1.29 per unit increase for SUVmax and 1.36 per unit increase for SUVpeak, independent of the number of lesions and the performance status.

### 3.4. Survival Estimates

**Figure 3:**
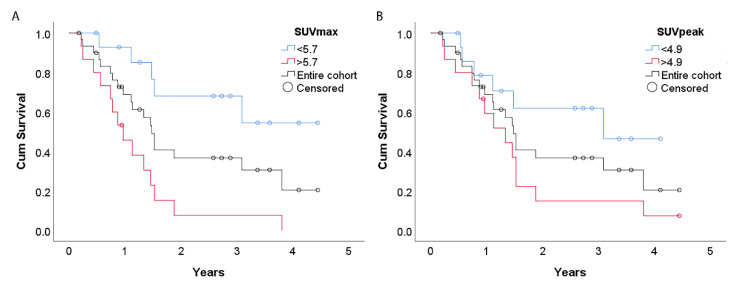
Survival curves with the cohort dichotomized at the median SUVmax (**A**) and median SUVpeak (**B**). The grey line represents the survival curve of the entire cohort. The estimated mean overall survival in patients with SUVmax > 5.7 g/mL was 14 months, and that for patients with SUVmax < 5.7 g/mL was 39 months (*p* < 0.001). For patients with SUVpeak > 4.9 g/mL, the estimated mean overall survival was 18 months, while for those with SUVpeak < 4.9 g/mL, it was 33 months (*p* = 0.04).

Median SUVmax and SUVpeak were 5.7 and 4.9 g/mL, respectively. The estimated mean overall survival in patients with SUVmax > 5.7 g/mL was 14 months, and that for patients with SUVmax < 5.7 g/mL was 39 months (*p* < 0.001). For patients with SUVpeak > 4.9 g/mL, the estimated mean overall survival was 18 months, while for those with SUVpeak < 4.9 g/mL, it was 33 months (*p* = 0.04) (Figure 3).

## 4. Discussion

In patients with STS, [^18^F]FDG-PET/CTs are often acquired for staging. Next to the identification of metastatic lesions, these scans provide quantitative information on the metabolic activity of the tumor tissue. The results in this study show that this biological characteristic has a prognostic value and turned out to be an independent predictor of overall survival in the soft tissue sarcoma patient group with metastatic disease. This information on tumor biology adds to the already known prognostic clinical parameters reported in the literature by Billingsley et al., Italiano et al., and Lochner et al., such as patient age, disease-free interval, number of lesions, FNCLCC grade, and histologic subtype [4,5,6].

In a systematic search that was conducted in preparation of this study, no report was found on the value of [^18^F]FDG-PET features in metastatic STS patients (Appendix A), while prognosis is especially relevant in a cohort where cure might not be the primary goal of treatment. The prognostic value of [^18^F]FDG-PET features in non-metastatic STS is studied more extensively. Original investigations focusing on this topic in patients with localized disease have found [^18^F]FDG-PET features to be significantly predictive for progression-free and overall survival [20,21,22,23]. Nevertheless, in some of these studies, the added value of the features is not corrected for clinical parameters, such as resectability of the tumor, neoadjuvant treatment, etc., leaving the effect of [^18^F]FDG-PET features difficult to interpret on an individual level. In studies performing multivariate analyses, results are variable and partly clouded due to the limited statistical power caused by small cohort sizes [24,25,26].

In the current study, the overall survival of the whole cohort was comparable with survival in recent larger studies, suggesting the current study population is representative, and our findings might add to the ability to accurately predict survival in patient with metastases from STS [4,5]. Our results show both SUVmax and SUVpeak to have prognostic value, and therefore, are in line with the results in patients with localized disease. For SUVmean, TLG, and MTV, however, studies in localized STS patients typically find significant correlations with overall survival, while no predictive value was found in the metastatic cohort in our study [22,23,25]. Partially, this could be caused by the limited cohort size. Another plausible reason for this discrepancy is found in the composition of these features and the biological background they resemble. All [^18^F]FDG-PET features investigated in this study, i.e., SUVmax, SUVpeak, SUVmean, TLG, and MTV, quantify the metabolism in selected tumor tissue, but SUVmean and inherently TLG and MTV are strongly dependent on tumor size next to metabolism and thus altered after resection of the primary tumor. In contrast, SUVmax and SUVpeak are not dependent on lesion size and thus resemble the metabolic potential of tumor cells accurately, even after surgical volume reduction. Thus, the results suggest that the prognosis of a metastatic STS patient is determined by the most aggressive tumor clone in the body.

Research in other tumor types, such as breast, colorectal, and lung carcinoma, also shows added prognostic value of [^18^F]FDG-PET features next to clinical parameters in cohorts of patients with metastasized disease [16,27,28]. In contrast with the current results, TLG and MTV generally also show a correlation to survival in these cohorts. An explanation for this discrepancy is the relative heterogeneous population in our study, including both patients with synchronous diagnosis of the primary tumor and metastasis and patients with diagnosis of metastasis after resection of the primary tumor. In addition, differences in tumor biology, such as pattern and interval of spread, might cause deviation between results in different tumor types.

A strength of this study is the use of a multivariate analysis to determine the added value of the PET features in addition to prognostic clinical parameters that are readily available. This multiparametric analysis showed that both SUVmax and SUVpeak provide prognostic value, next to the two strongest predictive clinical characteristics. Furthermore, the [^18^F]FDG-PET scans are often performed in standard clinical practice for staging of disease, and therefore, the features can be determined without extra costs and distress for the patients [7]. There are some limitations to this study. Due to the retrospective nature, the performance status of some patients could not be determined accurately. Moreover, the limited cohort size and the heterogeneity in tumor subtypes prohibited definitive conclusions about [^18^F]FDG-PET features when correcting for all known clinical parameters. In this regard, especially the link with the wide variety of histological subtype remains unexplored to some extent.

In larger studies investigating prognosis in metastatic STS patients, correlations with subtype are typically found [4,5,6]. These results are, however, partly contradicting regarding which subtypes are causing poor survival rates. With metastatic leiomyosarcoma as a reference, both Italiano et al. and Lochner et al. concluded that patients with metastatic undifferentiated soft tissue sarcoma or malignant peripheral nerve sheath tumors have an impaired survival but reported conflicting results regarding liposarcoma and synovial sarcoma patients [4,5]. This leads to the conclusion that a correlation between histologic subtype and survival in metastatic patients exists but is difficult to define. Several reasons for this complexity are rarity of subtypes, heterogeneity within sarcoma subtypes, and shifts in histologic definitions of subtypes over the years. In the current study, the biological differences between histologic subtypes might have amplified the predictive value of quantitative [^18^F]FDG-PET features on survival. In literature, relatively aggressive subtypes, such as undifferentiated soft tissue sarcoma, are found to show high FDG avidity. Other specific subtypes, such as (myxoid) liposarcomas, tend to show relatively low avidity [29,30]. Nevertheless, these studies report varying and non-specific SUVmax values within subtypes, suggesting [^18^F]FDG-PET features could provide additional prognostic information. Figure 2 presents examples of differences in SUVmax between and within STS subtypes. Future studies validating the prognostic value of quantitative [^18^F]FDG-PET features in metastatic STS patients should aim to address the link with histologic subtypes.

Furthermore, the use of multimodality imaging should be considered in research aiming to identify more prognostic biomarkers in patients with metastatic STS. Magnetic resonance (MR) imaging is widely used for the characterization of localized soft tissue tumors. Quantitative diffusion-weighted imaging (DWI) and dynamic contrast-enhanced (DCE) MR features are linked to tumor grade, response to treatment, and survival [31,32]. Multimodality imaging with [^18^F]FDG-PET/MR showed increased usefulness over [^18^F]FDG-PET alone in several studies on localized STS [33,34]. This raises the hypothesis that the addition of quantitative MR parameters to clinical and [^18^F]FDG-PET parameters could improve the characterization of tumor biology in patients with metastases from STS even further.

Personalized treatment in patients with metastases from STS is complex, and prognostic factors are important for multiple considerations during the development of treatment strategies. For example, factors linked to an impaired prognosis support the addition of chemotherapy to surgery in patients with resectable metastases. A high number of tumor lesions and a short recurrence-free interval are factors that are typically used for this purpose, as stated in the recent ESMO-EURACAN-GENTURIS guidelines [7]. The current study shows added value of [^18^F]FDG-PET features to these clinical factors. Moreover, in treatment strategies with a palliative intent specifically, periods without active treatment can be desirable to warrant the quality of life of patients. Prognostic factors are decisive in the timing of these treatment-free periods, as they are partly guided by the expected time to progression of disease [35].

## 5. Conclusions

In conclusion, personalized medicine is especially challenging and important in this patient group with strongly impaired survival rates. Therefore, accurate information about individual patient prognosis should be pursued before individual patient management decision making. Next to clinical and pathological characteristics, biological tumor characteristics such as metabolic parameters on [^18^F]FDG-PET scans can be considered for this purpose. In this regard, the current study finds SUVmax and SUVpeak to be significantly predictive for overall survival in patients with metastases from STS. Furthermore, both features add prognostic value to the best performing clinical parameters.

## Figures and Tables

**Table 1:** Patient characteristics expressed as mean and standard deviation, median and quartile range, or as number and percentages of the whole population. * FNCLCC grade such as reported in pathologic reports. For round cell sarcoma, rhabdomyosarcoma, angiosarcoma, and intima sarcoma, grade was not reported in pathologic reports. These highly aggressive tumors were categorized as grade 3 in this study. In one patient with a morphologic myxoid liposarcoma, no FNCLCC classification was performed (not applicable), and this patient was excluded from the univariate analysis for FNCLCC grade. † Morphologically, this tumor resembled a myxoid liposarcoma, but a characteristic translocation could not be demonstrated.

Characteristics, n = 31			
Age	59 ± 18	Histologic subtype	
Sex		Undifferentiated soft tissue sarcoma	8 (26%)
Male	20 (65%)	Myxofibrosarcoma	6 (19%)
Female	11 (35%)	MPNST	5 (16%)
WHO performance status		Leiomyosarcoma	3 (10%)
Unknown	8 (26%)	Dedifferentiated liposarcoma	2 (6%)
0	10 (32%)	Synovial sarcoma	2 (6%)
1	11 (35%)	Myxoid liposarcoma †	1 (3%)
2	2 (7%)	Round cell sarcoma	1 (3%)
Location of primary tumour		Rhabdomyosarcoma	1 (3%)
Lower extremity	18 (58%)	Angiosarcoma	1 (3%)
Upper extremity	3 (10%)	Intima sarcoma	1 (3%)
Trunk wall	3 (10%)	FNCLCC Grade *	
Deep trunk	6 (19%)	1	1 (3%)
Head/neck	1 (3%)	2	15 (48%)
Disease free interval		3	14 (45%)
Synchronous	7 (23%)	Not applicable	1 (3%)
<14 months	12 (39%)	Location of metastases	
>14 months	12 (39%)	Lung	7 (23%)
Number of lesions	3.3 ± 2.8	Lung and other	11(35%)
Sum of lesion diameters per patient (cm)	7.5 (6.0–17.5)	Soft tissue only	9 (29%)
		Bone only	4 (13%)

**Table 2:** Clinical variables and PET features in univariate Cox proportional hazard analyses.

Variable	Overall Survival		*p*-value
Clinical variables	Hazard ratio	95% CI	
Age (years)	1.02	0.99–1.04	0.2
Grade (3 versus 2)	1.26	0.50–3.21	0.6
Location (Deep trunk or upper extr. versus other)	0.91	0.34–2.40	0.8
Number of lesions WHO performance status (≥1 versus 0)	1.28 2.72	1.07–1.52 0.73–10.07	0.006 0.1
Disease free interval			0.2
Synchronous versus >14 months	3.36	0.94–12.0	
<14 months versus >14 months	1.44	0.47–4.47	
PET features			
SUVmax	1.16	1.05–1.29	0.004
SUVpeak	1.20	1.05–1.37	0.006
SUVmean	1.23	0.99–1.54	0.07
MTV	1.001	0.999–1.003	0.2
TLG	1.001	1.000–1.001	0.1

**Figure A1:**
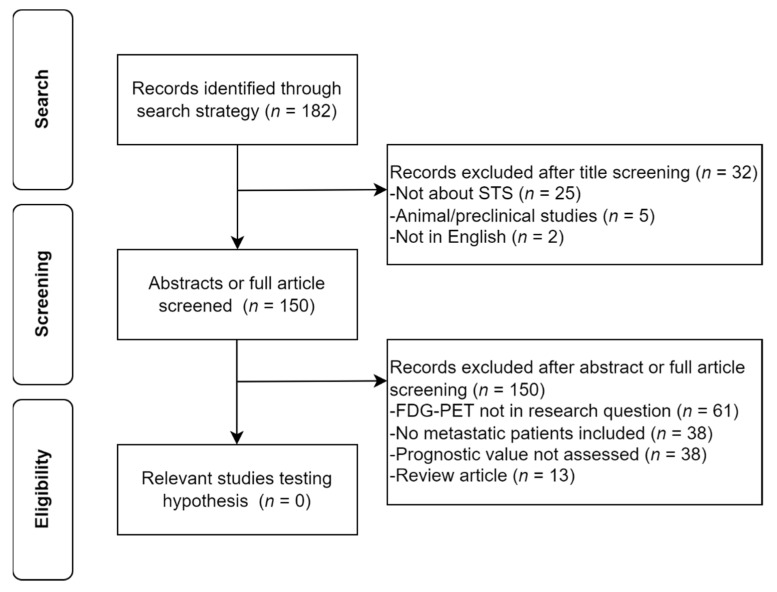
Flowchart of the performed literature search.

## Data Availability

The datasets generated during and/or analyzed during the current study are available from the corresponding author upon reasonable request.

## Appendix A. Literature Search

A systematic literature search was performed to identify available articles describing original research attending to our hypothesis. A search strategy based on our hypothesis was designed with the assistance of a medical librarian and conducted in PubMed on 1 September 2021. A total of 182 records were identified. After screening the titles, 32 records were excluded. In addition, after reading the abstracts and/or full articles, another 150 records were excluded. No articles that investigated our hypothesis were found (Figure A1).

Search strategy: ((“advanced soft tissue sarcoma”[tw] OR “advanced soft tissue sarcomas”[tw] OR “advanced sarcoma”[tw] OR “advanced sarcomas”[tw] OR “metastatic soft tissue sarcoma”[tw] OR “metastatic soft tissue sarcomas”[tw] OR “metastatic sarcoma”[tw] OR “metastatic sarcomas”[tw] OR “metastasized sarcoma”[tw] OR ((“soft tissue sarcomas”[tw] OR “soft tissue sarcoma”[tw] OR ((“Sarcoma”[Mesh:NoExp] OR “sarcoma”[tw] OR “sarcomas”[tw]) AND (“soft tissue”[tw] OR “soft tissues”[tw])) OR “Adenosarcoma”[mesh] OR “Carcinosarcoma”[mesh] OR “Fibrosarcoma”[mesh] OR “Hemangiosarcoma”[mesh] OR “Histiocytoma, Malignant Fibrous”[mesh] OR “Leiomyosarcoma”[mesh] OR “Liposarcoma”[mesh] OR “Lymphangiosarcoma”[mesh] OR “Mixed Tumor, Mesodermal”[mesh] OR “Myosarcoma”[mesh] OR “Myxosarcoma”[mesh] OR “Sarcoma, Alveolar Soft Part”[mesh] OR “Sarcoma, Clear Cell”[mesh] OR “Sarcoma, Myeloid”[mesh] OR “Sarcoma, Small Cell”[mesh] OR “Sarcoma, Synovial”[mesh] OR “Adenosarcoma”[tw] OR “Adenosarcomas”[tw] OR “Alveolar Soft Part Sarcoma”[tw] OR “Alveolar Soft Part Sarcomas”[tw] OR “Carcinosarcoma”[tw] OR “Carcinosarcomas”[tw] OR “Clear Cell Sarcoma”[tw] OR “Clear Cell Sarcomas”[tw] OR “Dermatofibrosarcoma”[tw] OR “Dermatofibrosarcomas”[tw] OR “Fibrosarcoma”[tw] OR “Fibrosarcomas”[tw] OR “Hemangiosarcoma”[tw] OR “Hemangiosarcomas”[tw] OR “Leiomyosarcoma”[tw] OR “Leiomyosarcomas”[tw] OR “Liposarcoma”[tw] OR “Liposarcomas”[tw] OR “Lymphangiosarcoma”[tw] OR “Lymphangiosarcomas”[tw] OR “Malignant Fibrous Histiocytoma”[tw] OR “Malignant Fibrous Histiocytomas”[tw] OR “Mesodermal Mixed Tumor”[tw] OR “Mesodermal Mixed Tumors”[tw] OR “Mesodermal Mixed Tumour”[tw] OR “Mesodermal Mixed Tumours”[tw] OR “Myeloid Sarcoma”[tw] OR “Myeloid Sarcomas”[tw] OR “Myosarcoma”[tw] OR “Myosarcomas”[tw] OR “Myxosarcoma”[tw] OR “Myxosarcomas”[tw] OR “Neurofibrosarcoma”[tw] OR “Neurofibrosarcomas”[tw] OR “Rhabdomyosarcoma”[tw] OR “Rhabdomyosarcomas”[tw] OR “Small Cell Sarcoma”[tw] OR “Small Cell Sarcomas”[tw] OR “Synovial Sarcoma “[tw] OR “Synovial Sarcomas”[tw] OR “Walker Carcinoma 256”[tw]) AND (“Neoplasm Metastasis”[Mesh] OR “Metastasis”[tw] OR “metasta*”[tw] OR “advanced”[tw]))) AND (“FDG-PET”[tw] OR “FDGPET”[tw] OR “18FDG-PET”[tw] OR “18FDGPET”[tw] OR “Positron-Emission Tomography”[Mesh] OR “Positron-Emission Tomography”[tw] OR “Positron-Emission”[tw] OR “PET”[tw] OR “PETCT”[tw] OR “Fluorodeoxyglucose F18”[Mesh] OR “Fluorodeoxyglucose F18”[tw] OR “FDG”[tw] OR “18F-FDG”[tw] OR “Fluorodeoxyglucose F 18”[tw] OR “Fludeoxyglucose F 18”[tw] OR “Fluorine 18 fluorodeoxyglucose”[tw] OR “18F Fluorodeoxyglucose”[tw] OR “18FDG”[tw] OR “2 Fluoro 2 deoxy D glucose”[tw] OR “2 Fluoro 2 deoxyglucose”[tw] OR “4-fluoro-4-deoxyglucose”[Supplementary Concept]) AND (“Mortality”[Mesh] OR “mortality”[Subheading] OR “Disease-Free Survival”[Mesh] OR “Survival Analysis”[Mesh] OR “Survival Rate”[Mesh] OR “Progression-Free Survival”[Mesh] OR “mortality”[tw] OR “survival”[tw] OR “death”[tw] OR “deaths”[tw] OR “Prognosis”[Mesh] OR “Prognosis”[tw] OR “prognos*”[tw] OR “outcome”[tw] OR “outcomes”[tw]) NOT ((“Case Reports”[ptyp] OR “case report”[ti]) NOT (“Clinical Study”[ptyp] OR “trial”[ti] OR “RCT”[ti]))).

## References

[1] Society A.C. (2017). Cancer Facts & Figures 2017.

[2] Callegaro D., Miceli R., Bonvalot S., Ferguson P., Strauss D.C., Levy A., Griffin A., Hayes A.J., Stacchiotti S., Pechoux C.L. (2016). Development and external validation of two nomograms to predict overall survival and occurrence of distant metastases in adults after surgical resection of localised soft-tissue sarcomas of the extremities: A retrospective analysis. Lancet Oncol..

[3] Acem I., Verhoef C., Rueten-Budde A.J., Grunhagen D.J., van Houdt W.J., van de Sande M.A., Aston W., Bonenkamp H., Desar I.M., Ferguson P.C. (2020). Age-related differences of oncological outcomes in primary extremity soft tissue sarcoma: A multistate model including 6260 patients. Eur. J. Cancer.

[4] Lochner J., Menge F., Vassos N., Hohenberger P., Kasper B. (2020). Prognosis of Patients with Metastatic Soft Tissue Sarcoma: Advances in Recent Years. Oncol. Res. Treat..

[5] Italiano A., Mathoulin-Pelissier S., Cesne A.L., Terrier P., Bonvalot S., Collin F., Michels J.J., Blay J.Y., Coindre J.M., Bui B. (2011). Trends in survival for patients with metastatic soft-tissue sarcoma. Cancer.

[6] Billingsley K.G., Lewis J.J., Leung D.H., Casper E.S., Woodruff J.M., Brennan M.F. (1999). Multifactorial analysis of the survival of patients with distant metastasis arising from primary extremity sarcoma. Cancer.

[7] Gronchi A., Miah A.B., Dei Tos A.P., Abecassis N., Bajpai J., Bauer S., Biagini R., Bielack S., Blay J.Y., Bolle S. (2021). Soft tissue and visceral sarcomas: ESMO-EURACAN-GENTURIS Clinical Practice Guidelines for diagnosis, treatment and follow-up ☆. Ann. Oncol..

[8] Van Glabbeke M., van Oosterom A.T., Oosterhuis J.W., Mouridsen H., Crowther D., Somers R., Verweij J., Santoro A., Buesa J., Tursz T. (1999). Prognostic factors for the outcome of chemotherapy in advanced soft tissue sarcoma: An analysis of 2,185 patients treated with anthracycline-containing first-line regimens—A European Organization for Research and Treatment of Cancer Soft Tissue and Bone Sarcoma Group Study. J. Clin. Oncol..

[9] Rueten-Budde A.J., van Praag V.M., van de Sande M.A., Fiocco M., Group P.S. (2021). External validation and adaptation of a dynamic prediction model for patients with high-grade extremity soft tissue sarcoma. J. Surg. Oncol..

[10] Rueten-Budde A.J., Van Praag V.M., Jeys L.M., Laitinen M.K., Pollock R., Aston W., van der Hage J.A., Dijkstra P.S., Ferguson P.C., Griffin A.M. (2018). Dynamic prediction of overall survival for patients with high-grade extremity soft tissue sarcoma. Surg. Oncol..

[11] Kalisvaart G.M., Bloem J.L., Bovee J., van de Sande M.A., Gelderblom H., van der Hage J.A., Hartgrink H.H., Krol A.D., de Geus-Oei L.F., Grootjans W. (2021). Personalising sarcoma care using quantitative multimodality imaging for response assessment. Clin. Radiol..

[12] Annovazzi A., Rea S., Zoccali C., Sciuto R., Baldi J., Anelli V., Petrongari M.G., Pescarmona E., Biagini R., Ferraresi V. (2020). Diagnostic and Clinical Impact of 18F-FDG PET/CT in Staging and Restaging Soft-Tissue Sarcomas of the Extremities and Trunk: Mono-Institutional Retrospective Study of a Sarcoma Referral Center. J. Clin. Med..

[13] Vlenterie M., Oyen W.J., Steeghs N., Desar I.M., Verheijen R.B., Koenen A.M., Grootjans W., De Geus-Oei L.F., Van Erp N.P., Van Der Graaf W.T. (2019). Early Metabolic Response as a Predictor of Treatment Outcome in Patients with Metastatic Soft Tissue Sarcomas. Anticancer Res..

[14] Reyes Marles R.H., Navarro Fernandez J.L., Puertas Garcia-Sandoval J.P., Santonja Medina F., Mohamed Salem L., Frutos Esteban L., Contreras Gutierrez J.F., Castellon Sanchez M.I., Ruiz Merino G., Claver Valderas M.A. (2021). Clinical value of baseline ^18^F-FDG PET/CT in soft tissue sarcomas. Eur. J. Hybrid Imaging.

[15] Chen L., Wu X., Ma X., Guo L., Zhu C., Li Q. (2017). Prognostic value of 18F-FDG PET-CT-based functional parameters in patients with soft tissue sarcoma: A meta-analysis. Medicine.

[16] De Geus-Oei L.F., Wiering B., Krabbe P.F., Ruers T.J., Punt C.J., Oyen W.J. (2006). FDG-PET for prediction of survival of patients with metastatic colorectal carcinoma. Ann. Oncol..

[17] Trojani M., Contesso G., Coindre J.M., Rouesse J., Bui N.B., de Mascarel A., Goussot J.F., David M., Bonichon F., Lagarde C. (1984). Soft-tissue sarcomas of adults; study of pathological prognostic variables and definition of a histopathological grading system. Int. J. Cancer.

[18] Boellaard R., Delgado-Bolton R., Oyen W.J., Giammarile F., Tatsch K., Eschner W., Verzijlbergen F.J., Barrington S.F., Pike L.C., Weber W.A. (2015). FDG PET/CT: EANM procedure guidelines for tumour imaging: Version 2.0. Eur. J. Nucl. Med. Mol. Imaging.

[19] Frings V., van Velden F.H., Velasquez L.M., Hayes W., van de Ven P.M., Hoekstra O.S., Boellaard R. (2014). Repeatability of metabolically active tumor volume measurements with FDG PET/CT in advanced gastrointestinal malignancies: A multicenter study. Radiology.

[20] Singh T.P., Sharma A., Sharma A., Bakhshi S., Patel C., Pandey A.K., Dhamija E., Batra A., Kumar R. (2021). Utility of 18F-FDG-PET/CT in management and prognostication of treatment naive late-stage soft tissue sarcomas. Nucl. Med. Commun..

[21] Fuglø H.M., Jørgensen S.M., Loft A., Hovgaard D., Petersen M.M. (2012). The diagnostic and prognostic value of ¹⁸F-FDG PET/CT in the initial assessment of high-grade bone and soft tissue sarcoma. A retrospective study of 89 patients. Eur. J. Nucl. Med. Mol. Imaging.

[22] Lee J.W., Heo E.J., Moon S.H., Lee H., Cheon G.J., Lee M., Kim H.S., Chung H.H. (2016). Prognostic value of total lesion glycolysis on preoperative ^18^F-FDG PET/CT in patients with uterine carcinosarcoma. Eur. Radiol..

[23] Chang K.J., Lim I., Park J.Y., Jo A.R., Kong C.B., Song W.S., Jo W.H., Lee S.Y., Koh J.S., Kim B.I. (2015). The Role of ^18^F-FDG PET/CT as a Prognostic Factor in Patients with Synovial Sarcoma. Nucl. Med. Mol. Imaging.

[24] Lisle J.W., Eary J.F., O’Sullivan J., Conrad E.U. (2009). Risk assessment based on FDG-PET imaging in patients with synovial sarcoma. Clin. Orthop. Relat. Res..

[25] Choi E.S., Ha S.G., Kim H.S., Ha J.H., Paeng J.C., Han I. (2013). Total lesion glycolysis by 18F-FDG PET/CT is a reliable predictor of prognosis in soft-tissue sarcoma. Eur. J. Nucl. Med. Mol. Imaging.

[26] Baum S.H., Frühwald M., Rahbar K., Wessling J., Schober O., Weckesser M. (2011). Contribution of PET/CT to prediction of outcome in children and young adults with rhabdomyosarcoma. J. Nucl. Med..

[27] Satoh Y., Nambu A., Ichikawa T., Onishi H. (2014). Whole-body total lesion glycolysis measured on fluorodeoxyglucose positron emission tomography/computed tomography as a prognostic variable in metastatic breast cancer. BMC Cancer.

[28] Lim Y., Bang J.I., Han S.W., Paeng J.C., Lee K.H., Kim J.H., Kang G.H., Jeong S.Y., Park K.J., Kim T.Y. (2017). Total lesion glycolysis (TLG) as an imaging biomarker in metastatic colorectal cancer patients treated with regorafenib. Eur. J. Nucl. Med. Mol. Imaging.

[29] Macpherson R.E., Pratap S., Tyrrell H., Khonsari M., Wilson S., Gibbons M., Whitwell D., Giele H., Critchley P., Cogswell L. (2018). Retrospective audit of 957 consecutive ^18^F-FDG PET-CT scans compared to CT and MRI in 493 patients with different histological subtypes of bone and soft tissue sarcoma. Clin. Sarcoma Res..

[30] Charest M., Hickeson M., Lisbona R., Novales-Diaz J.A., Derbekyan V., Turcotte R.E. (2009). FDG PET/CT imaging in primary osseous and soft tissue sarcomas: A retrospective review of 212 cases. Eur. J. Nucl. Med. Mol. Imaging.

[31] Spinnato P., Kind M., Le Loarer F., Bianchi G., Colangeli M., Sambri A., Ponti F., van Langevelde K., Crombe A. (2021). Soft Tissue Sarcomas: The Role of Quantitative MRI in Treatment Response Evaluation. Acad. Radiol..

[32] Casali P.G., Abecassis N., Aro H.T., Bauer S., Biagini R., Bielack S., Bonvalot S., Boukovinas I., Bovee J., Brodowicz T. (2018). Soft tissue and visceral sarcomas: ESMO-EURACAN Clinical Practice Guidelines for diagnosis, treatment and follow-up. Ann. Oncol..

[33] Chodyla M., Demircioglu A., Schaarschmidt B.M., Bertram S., Morawitz J., Bauer S., Podleska L., Rischpler C., Forsting M., Herrmann K. (2021). Evaluation of the Predictive Potential of 18F-FDG PET and DWI Data Sets for Relevant Prognostic Parameters of Primary Soft-Tissue Sarcomas. Cancers.

[34] Chodyla M., Demircioglu A., Schaarschmidt B.M., Bertram S., Bruckmann N.M., Haferkamp J., Li Y., Bauer S., Podleska L., Rischpler C. (2021). Evaluation of ^18^F-FDG PET and DWI Datasets for Predicting Therapy Response of Soft-Tissue Sarcomas Under Neoadjuvant Isolated Limb Perfusion. J. Nucl. Med..

[35] Dangoor A., Seddon B., Gerrand C., Grimer R., Whelan J., Judson I. (2016). UK guidelines for the management of soft tissue sarcomas. Clin. Sarcoma Res..

